# Biomimetic Chromatographic Studies Combined with the Computational Approach to Investigate the Ability of Triterpenoid Saponins of Plant Origin to Cross the Blood–Brain Barrier

**DOI:** 10.3390/ijms22073573

**Published:** 2021-03-30

**Authors:** Katarzyna Stępnik

**Affiliations:** Department of Physical Chemistry, Institute of Chemical Sciences, Faculty of Chemistry, Maria Curie–Sklodowska University in Lublin, 20-031 Lublin, Poland; katarzyna.stepnik@umcs.pl

**Keywords:** triterpenoid saponins, computational studies, Quantitative Structure-Activity Relationship (QSAR), biomimetic studies, micellar liquid chromatography, IAM stationary phase

## Abstract

Biomimetic (non-cell based in vitro) and computational (in silico) studies are commonly used as screening tests in laboratory practice in the first stages of an experiment on biologically active compounds (potential drugs) and constitute an important step in the research on the drug design process. The main aim of this study was to evaluate the ability of triterpenoid saponins of plant origin to cross the blood–brain barrier (BBB) using both computational methods, including QSAR methodology, and biomimetic chromatographic methods, i.e., High Performance Liquid Chromatography (HPLC) with Immobilized Artificial Membrane (IAM) and cholesterol (CHOL) stationary phases, as well as Bio-partitioning Micellar Chromatography (BMC). The tested compounds were as follows: arjunic acid (*Terminalia arjuna*), akebia saponin D (*Akebia quinata*), bacoside A (*Bacopa monnieri*) and platycodin D (*Platycodon grandiflorum*). The pharmacokinetic BBB parameters calculated in silico show that three of the four substances, i.e., arjunic acid, akebia saponin D, and bacoside A exhibit similar values of brain/plasma equilibration rate expressed as logPS_Fubrain_ (the average logPS_Fubrain_: −5.03), whereas the logPS_Fubrain_ value for platycodin D is –9.0. Platycodin D also shows the highest value of the unbound fraction in the brain obtained using the examined compounds (0.98). In these studies, it was found out for the first time that the logarithm of the analyte–micelle association constant (logK_MA_) calculated based on Foley’s equation can describe the passage of substances through the BBB. The most similar logBB values were obtained for hydrophilic platycodin D, applying both biomimetic and computational methods. All of the obtained logBB values and physicochemical parameters of the molecule indicate that platycodin D does not cross the BBB (the average logBB: −1.681), even though the in silico estimated value of the fraction unbound in plasma is relatively high (0.52). As far as it is known, this is the first paper that shows the applicability of biomimetic chromatographic methods in predicting the penetration of triterpenoid saponins through the BBB.

## 1. Introduction

Neurodegenerative related diseases constitute a growing health issue in aging populations worldwide. It is estimated that the number of patients suffering from Alzheimer’s disease alone was more than 35 million in 2012 and it will double by 2030 and more than triple by 2050 [1]. Therefore, one of the key research requirements is to look for substances capable of crossing the blood–brain barrier (BBB) and those that may have neuroprotective properties.

In the drug discovery process, the main goal is to explore and optimize therapeutic agents with desirable pharmacodynamic, pharmacokinetic and toxicological properties. For this purpose, predictive tools for accurate assessment of these properties are useful especially in the early development stages in the drug discovery process [2,3,4,5]. The need for high-throughput screening has increased dramatically over the past several decades as a result of constant pressure on pharmaceutical companies to accelerate drug discovery while reducing drug development costs [3]. In this aspect, an important role is played by the biomimetic (non-cell based in vitro) and computational (in silico) methods which have been developed and improved in recent years for the prediction of compound ADME properties (absorption, distribution, metabolism, excretion) [6,7].

Nevertheless, the prediction of biological activity in itself, connected with estimation of ADME and toxicity parameters, should be based on a spectrum of in vivo, in vitro and in silico methods. In relation to the purpose of the presented studies, the experimental in vivo determination of blood–brain barrier permeability seems to be very difficult mainly because it requires complex techniques and is usually expensive and time-consuming [8,9,10]. Therefore, in vivo studies should be preceded by alternative tests including computational, biomimetic and neurotoxicity assays [3,4]. Such an approach is especially important because of reagent saving (Green Chemistry principles) and protection of animals used for scientific purposes (the European Community Council Directive for the Care and Use of laboratory animals of 22 September 2010 (2010/63/EU)). The Directive is firmly based on the principle of the Three Rs, to replace, reduce and refine the exploitation of animals for scientific purposes. The main aim of the European Centre for the Validation of Alternative Methods (ECVAM), a unit of the Institute for Health and Consumer Protection of the European Commission’s Joint Research Centre (JRC), is active supporting of alternative methods to those in vivo, which could replace, reduce and refine the use of laboratory animals [11].

The main aim of the study was to evaluate the ability of naturally occurring triterpenoid saponins to cross the blood–brain barrier based on both computational analyses including QSAR methodology and biomimetic studies using adequate liquid chromatographic techniques. These studies precede in vivo experiments which can be the subject of further studies. The tested compounds were as follows: arjunic acid (*Terminalia arjuna*), akebia saponin D (*Akebia quinata*), bacoside A (*Bacopa monnieri*) and platycodin D (*Platycodon grandiflorum*).

There are a few papers on the permeability of the tested triterpenoid saponins through the BBB; however, the studies were not based on both in silico and non-cell based in vitro methods [12,13,14,15,16,17,18]. Triterpenoid saponins were selected for this research because they have a broad spectrum of biological activity. For example, numerous bioactive saponins isolated from *Terminalia arjuna* (*Combreatceae*) have been reported to have antioxidant, anti-ischemic, antihypertensive, antihypertrophic and other effects [19,20,21,22,23,24,25,26,27]. Due to their pharmacological properties, they are used in pharmacy and medicine. In the presented studies the applicability of the biomimetic chromatographic methods in predicting the BBB-permeation potential of triterpenoid saponins was shown for the first time. 

In recent years there has also been an increase in interest in the non-cell based in vitro methods including chromatographic methods, that do not use the real cell lines but whose systems (mobile or stationary phase) provide a simple model of the biological barrier. The following methods play an important role in this regard i.e., Bio-partitioning Micellar Chromatography (BMC), High Performance Liquid Chromatography (HPLC) with the Immobilized Artificial Membrane (IAM), and cholesterol-bonded-silica stationary phase (CHOL). 

The unique properties of the microvasculature of the central nervous system (CNS) are characterized by the concept of the blood-brain barrier (BBB) [28]. The BBB, composed of many cell types, is the dynamic interface between the blood and the brain tissue whose task is to maintain the tightly controlled microenvironment of the brain [29]. This is a diffusion barrier that is essential for the normal functioning of the central nervous system [30]. In other words, due to the BBB’s endothelial cells, the brain is partitioned from the peripheral circulation, thus regulating CNS homeostasis providing the flux of relevant substances from the bloodstream, e.g., oxygen and glucose, and protecting the CNS against neurotoxins, inflammation, injuries and diseases [28,31]. 

Both the paracellular (between the adjacent cells) and transcellular (through the cells) pathways can occur in the passage of molecules across the BBB endothelium cells [32]. The paracellular pathway is followed by small, usually hydrophilic molecules and ions in accordance with their size and charge [33]. It is difficult for such molecules to partition into the cell membranes, therefore they cannot cross the BBB via the transcellular pathway [34]. These substances can simply diffuse between the adjacent cells down their concentration gradient; however, the diffusion is limited by the presence of tight junctions placed at the outermost end of the intercellular space [33]. Instead, the small hydrophobic drugs favor the transcellular pathway because they can partition into the cell membranes [34]. Different mechanisms are involved in the transcellular pathway, i.e., the passive diffusion of lipophilic compounds, receptor-mediated shuttling, and transcytosis [35,36]. Some molecules such as oxygen, CO_2_, alcohol, and steroid hormones can freely penetrate the BBB transcellularly by diffusion and dissolving in the lipid plasma membrane [37,38]. There are also the other mechanisms, i.e., the specific receptor mediated or vesicular mechanisms which are used by almost all other substances [37]. Hydrophilic molecules may enter the brain using specific transport mechanisms [39]. 

The endothelial cells of brain microvasculature which tend to form tight junctions are the anatomical constituents of the BBB. The neurovascular unit are, in turn, constructed by the endothelial cells with pericytes, astrocytes, oligodendrocytes, microglia, and neurons [40]. It is also worth emphasizing that a key role in the development of microcirculation is played by pericytes, being embedded in the basement membrane of brain capillaries [41,42].

The bulk of the current computational and biomimetic methods of determination of a substance permeability through the BBB is based on a pharmacokinetic parameter, i.e., the brain/blood partitioning in the steady state, expressed as logBB. This parameter is defined as the logarithmic ratio between the concentration of a substance in brain and its concentration in blood [43]:

logBB = log(conc. in brain/conc. in blood)(1)

In the early stages of the neuroprotective drug discovery process, it is necessary to study the ability of various substances to cross the blood–brain barrier. This property should be treated as a key factor for further research including in vivo [44]. In this investigation the relationships between the logBB values and various partition indices were examined. This is crucial to be able to compare their possible effectiveness in describing the BBB passage.

Bio-partitioning chromatography is a mode of micellar liquid chromatography (MLC) in which a mobile phase is composed of non-ionic surfactant—poly-oxyethylene (23) lauryl ether, Brij35, above the critical micellar concentration (CMC) in order to form micelles [45,46]. The Brij35 micelle is assumed to be a kind of simple, chemical model of the bio-membrane. This composition of the chromatographic system makes BMC useful in modelling various biological behaviour of different kinds of drug [47,48,49,50,51,52,53]. The applicability of BMC in predicting biological activity of compounds can be attributed to the similarities between the BMC systems and biological barriers as well as extracellular fluid [47]. Therefore, the retention of a compound in BMC reflects appropriately the bio-partitioning process, i.e., the solute partitioning into the lipid bilayers of biological membranes [52]. 

The retention of compounds in the micellar chromatography depends on the type of interactions (electrostatic and/or hydrophobic), with a surfactant-modified stationary phase and micelles [54,55,56,57]. For the ionic compounds, both interactions should be considered [48]. On the other hand, retention of a substance depends also on the properties of the molecule itself, i.e., its lipophilic, steric and electronic properties, being the most important parameters governing the transport and drug–receptor interactions [58]. 

Due to the everlasting development of dynamic combinatorial/covalent chemistry (DCC), it is possible to generate a wide range of structurally diverse compounds through a systematic, repetitive and covalent combination of various “building blocks” [59]. Both small molecule receptor binders and larger biomimetic macromolecules can be produced using the DCC technique with particular three-dimensional structural architectures [60]. More realistic biomimetic chromatographic models than the theoretical one are the Immobilized Artificial Membrane (IAM), as well as cholesterol immobilized on silica, based on the main constituents of eukaryotic cell membranes, i.e., analogues of phosphatidylcholine (PC) and cholesterol (CHOL), respectively [61,62]. Both, IAM and CHOL systems are increasingly used to study the biological properties of different organic compounds [63].

The retention in the IAM stationary phases results from lipophilic, electrostatic and other secondary interactions, contrary to the traditional n-octanol-water partitioning behaviour [64]. The IAM retention factors are often correlated with different kinds of biological activity and pharmacokinetic properties including ecotoxicity [65], blood-brain barrier absorption [61], bioconcentration [66], oral absorption [67], transdermal transport [68], volume of distribution [69,70], protein binding properties [69], and brain penetration [71]. Table 1 shows the important areas of biomimetic chromatographic methods applications used in this research. 

## 2. Results

### 2.1. Division of the Dataset for the QSAR Studies

The chemical structures of the investigated triterpenoid saponins are presented in Table 2.

The QSAR model used here (Equation (2)) was established based on my former studies to calculate the logBB values for the different group of triterpenoid saponins. Therefore, there was a reasonable assumption that the previously used model would be adequate for the currently tested compounds. The exact procedure for selecting and dividing the dataset for the QSAR analysis was precisely described in the previous paper [90].

Briefly, in order to establish a new QSAR model, the dataset comprised 40 chemically diverse compounds with corresponding experimentally determined logBB values [91] (Table S1). Among the tested compounds, 10 were selected to form the test set, whereas 30 compounds were chosen to be a training set. The division of the dataset was made several times in a random manner. The tested saponins were not used to develop the model. They were external to the model. To establish the model, multiple linear regression (MLR) methodology with backward elimination of variables was applied. This procedure was aimed at reducing the differences between the actual and estimated logBB values. The established QSAR model examines the quantitative relationship between the structure of a molecule and its ability to cross the BBB, expressed as logBB. In other words, various physicochemical parameters were determined to correlate specific properties of a molecule with its ability to cross the BBB (Table S2). Many attempts were made to obtain the best fit between the logBB values and the physicochemical properties of molecules. Each time analysis of variance was made. This was based on the following parameters: the determination coefficient (R^2^), predicted residual sum of squares (PRESS), root-mean-square error (RMSE), and root-mean-square error of leave-ten-out cross-validation (RMSECV). The leave-ten-out (LTO) cross-validation procedure was used to assess the predictive potency of the model. Then, to evaluate the reliability of the model, analysis of variance was made and the applicability domain (AD) was applied [90].

To calculate the logBB values for the tested saponins, the QSAR model previously obtained was used [90]. In this QSAR predictive model the BBB penetration potential was correlated with the lipophilic properties (logPow), excess molar refraction (E), the difference between the logarithms of n-octanol/water and cyclohexane/water partition coefficients values (ΔlogP) being the hydrogen-bonding potential:

logBB = −0.114 − 0.098 ΔlogP + 0.278 logPow + 0.218E(2)

In the above equation, n = 40, R^2^_CV_ = 78.25%, R^2^_pred_ = 74.02%, and S = 0.436.

### 2.2. BBB Descriptors Calculated In Silico

The values of the most important BBB pharmacokinetic descriptors of the brain were determined using the ACD/Percepta software, i.e., logBB, the distribution of a substance in the blood-brain area (the BBB penetration descriptor); logPS, the rate of passive diffusion/permeability (the permeability-surface area product); logPS_Fubrain_, the brain/plasma equilibration rate; Fu, the fraction unbound in plasma; and Fb, the fraction unbound in brain (Table 3).

In a further part of the in silico studies, other significant physicochemical values were calculated in a similar way. According to the Hansch approach [58,92], steric, electronic and lipophilic parameters of the molecules were determined. The steric parameters describe the geometry of the molecule and, in particular, the overall size and shape of the molecule, demonstrating the potential fit of a compound to its cellular target. Among the steric parameters, the molar volume and molecular weight (MW) of the tested saponins were determined. The lipophilic parameters—crucial from the point of view of the undertaken scientific activity—describe the ability of a compound to penetrate the biological membranes (including the BBB) and thus characterize the transport and resorptive properties of a compound. The following logarithms of partition coefficients were determined: n-octanol/water (logPow), heptane/water (logPhw) and cyclohexane/water (logPcw).

Moreover, the excess molar refraction (E) was determined in silico based on the linear free energy relationship (LFER) methodology originally employed by Abraham [93,94]. It is commonly known that the LFER theory has been successfully used to characterize various biological and physicochemical processes, including permeability of a substance through biological membranes. As shown in the previous paper [90], the excess molar refraction taken from the LFER approach, combined with the lipophilic descriptor logPow and the hydrogen-bonding parameter ΔlogP, provide a promising combination to estimate the logBB values of triterpenoid saponins. In addition, other important physicochemical parameters have been calculated in silico (ACD/Percepta software), i.e., the topological polar surface area (TPSA), and polarizability, which can also determine the ability of the molecule to cross biological barriers, including the BBB [95]. Some of these parameters are presented in Table 4.

### 2.3. Chromatographic Biomimetic Studies

Chromatographic biomimetic methods, including IAM, BMC, and CHOL, were used to determine BBB permeability of the tested triterpenoid saponins. For this purpose, the values of logkw, being the logarithm of retention factor extrapolated to pure water, were determined. These values were compared with the logBB values calculated in silico and those obtained using the QSAR model (Equation (2)). Logkw is recognized to be an alternative to the logPow lipophilicity descriptor [96]. On the other hand, lipophilicity is one of the most important features influencing the ability of substances to cross biological barriers [58].

Moreover, for the micellar biomimetic studies (BMC), Foley’s equation [97] (Equation (3)) was used to calculate important physicochemical parameters, i.e., the analyte–micelle association constant (K_MA_) and the partition coefficient of the analyte between the stationary phase and water (Psw). These parameters can describe possible interactions in the micellar system since the Brij35 micelle can be treated as a simple model of a biological barrier.

(3) $$\frac{1}{k}=\;\frac{K_{\mathrm{MA}}}{P_{\mathrm{SW}}\Phi }C_{M}+\;\frac{1}{P_{\mathrm{SW}}\Phi }$$

where k is the retention factor, C_M_ is the concentration of micelles, K_MA_ is the analyte–micelle association constant, P_SW_ is the partition coefficient of the analyte between the stationary phase and water, and Φ is the volume ratio of the stationary phase to the mobile phase volume.

In the research the following thesis was put forward for the first time: the logarithm of the analyte–micelle association constant (logK_MA_) values obtained from the bio-partitioning micellar systems corresponds to the logBB values. Thus, this indicates that the logBB values can be pre-estimated based only on the BMC retention data. LogK_MA_ can be a useful tool for rapid assessment of the ability of a substance to cross the BBB, especially in the early stage of research. The obtained results seem to confirm this thesis.

**Figure 1:**
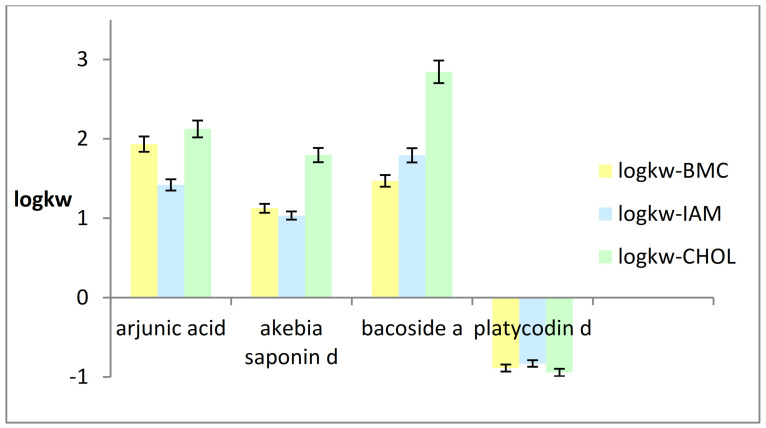
Logkw-BMC, logkw-IAM, and logkw-CHOL values obtained from the tested systems.

The obtained values of logarithm of retention factor extrapolated to pure water by means of the BMC, IAM, and CHOL methods (logkw-BMC, logkw-IAM, logkw-CHOL, respectively) are presented in Figure 1.

**Figure 2:**
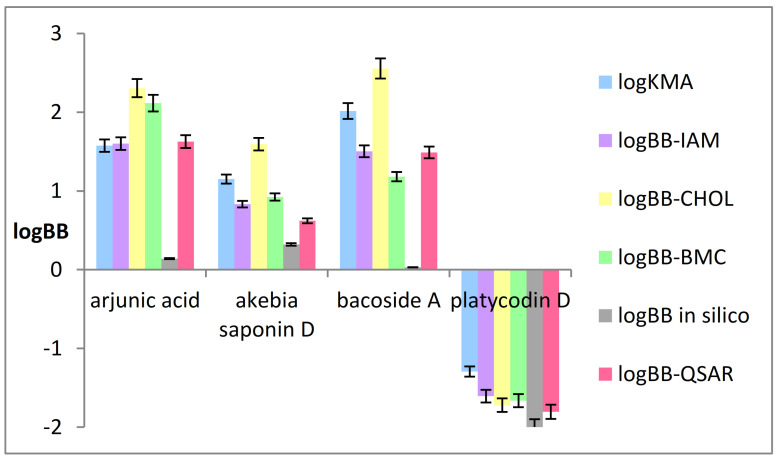
LogBB values obtained using the biomimetic and computational methods.

As mentioned above the logkw parameter, obtained from the biomimetic chromatographic systems, is recognized to be an alternative to the logarithm of the n-octanol/water partition coefficient (logPow) lipophilicity descriptor. Therefore, logkw-BMC, logkw-IAM, and logkw-CHOL values were used for the calculation of logBB based on the QSAR equation (Equation (2)), instead of the logPow parameter. The values of logBB-BMC, logBB-IAM, and logBB-CHOL were thus calculated. In Figure 2 the above-mentioned logBB values and logBB in silico calculated using the ACD/Percepta software, as well as those of logK_MA_ that can be treated as an equivalent to the logBB parameter, are presented.

## 3. Discussion

The plant derived secondary metabolites including triterpenoid saponins have proved to be interesting sources of compounds with neuroprotective properties e.g., onjisaponins isolated from the roots of *Polygala tenuifolia* [98], platycodins from *Platycodi* radix [99] or medicagosides A-F from *Medicago sativa* L. [100]. Many saponins demonstrate therapeutic efficacy. In most cases they can cross the blood–brain barrier and can affect the central nervous system (CNS) including nerve cells of the brain and spinal cord which tend to maintain many direct body functions. They can also affect the autonomic nervous system which, in turn, is responsible for regulating, among others, heartbeat, blood circulation and breathing.

In the investigations, it was observed that the time to reach brain equilibrium can be prolonged when the BBB permeability–surface area product (PS) or the fraction unbound in the brain (Fb) decreases [101]. However, this time value did not change when the brain/plasma equilibration rate (PS_Fubrain_) was kept constant or when PS decreased and Fb increased simultaneously. Therefore, the compounds having similar PS_Fubrain_ values should exhibit comparable time to reach brain equilibrium, although they may have a much different PS value. In the experiment, three of the four substances, i.e., arjunic acid, akebia saponin D, and bacoside A exhibit similar logPS_Fubrain_ values (the average logPS_Fubrain_: −5.03), whereas the logPS_Fubrain_ value for platycodin D is −9.0. Moreover, platycodin D also shows the highest value of the unbound fraction in the brain among the examined compounds (0.98).

Based on the obtained values of pharmacokinetic BBB parameters it can be concluded that among the tested compounds, only platycodin D is probably not permeable through the BBB (logBB from −2 to −1.29), even though the in silico estimated value of the fraction unbound in plasma is relatively high (0.52).

Nevertheless, there are reports in the literature [102] where it is assumed that platycodin D can permeate the BBB. However, the authors did not investigate the BBB permeability of this compound directly but based their assumption on the results of the studies of three other compounds capable of penetrating the BBB, i.e., saikosaponin A, glycyrrhizin, and ginsenoside. These compounds could attenuate neuroinflammation in the brain and have the ability to penetrate the blood–brain barrier [103]. There are also significant differences between the physicochemical parameters of platycodin D and the above-mentioned compounds, especially those steric (MW) and lipophilic (logPow) ones. For comparison, the molecular weights (MW) for saikosaponin A, glycyrrhizin, and ginsenoside are as follows: 781, 822.9, 785 g/mol, respectively, and MW for platycodin D is 1225.32 g/mol, whereas logPow values are equal to: 2.5, 3.7, 4, respectively, and for platycodin D,−3.7. As one can see, platycodin D has a much larger molecule, and, above all, it is a hydrophilic compound, unlike the above-mentioned substances. These characteristics significantly condition the permeation of compounds through biological barriers. Due to the specificity of the blood–brain barrier, only molecules with higher lipophilicity and lower molecular weight can enter the membrane easily [104].

Nevertheless, the research proves that platycodin D has a definite effect on the CNS, e.g., it protects the hippocampal CA1 region pyramidal neurons from an ischemic damage, blocks the activation of glial cells, and significantly reduces the neuroinflammation induced by ischemia/reperfusion in the hippocampal CA1 region [105]. Moreover, it has been proved that platycodin D could improve ethanol-induced memory impairment in mice [106].

Based on the studies presented in this paper, it is largely probable that the other three saponins, i.e., arjunic acid, akebia saponin D, and bacoside A, can penetrate the BBB. For these substances a similar brain-plasma equilibration rate was obtained (logPS; the average: −3.73). There are several scientific reports in the literature that confirm the ability of the saponins to cross the BBB. To study the neuroprotective effect of saponins occurring in the *Terminalia chebula* Retz, arjunic acid among others, the brain tissues of rats were analysed after the intragastric administration of the extract. The absorbed components in the rat plasma and brain were detected and analyzed using ultra-performance liquid chromatography–quadrupole time-of-flight mass spectrometry (UPLC–QTOF-MS) [107]. The postmortem studies of brain tissues showed that arjunic acid is present in rat brain tissues. Therefore, this confirms the ability of arjunic acid to cross the BBB.

Pro-cognitive properties of akebia saponin D were analyzed using in vivo behavioral tests on rats. Emotional disturbances and impairment i.e., anxiety, depression, or memory deficits, were induced by the intracerebroventricular injection of amyloid β-peptide (Aβ25-35) into the lateral ventricles to simulate the symptoms of Alzheimer’s disease (AD) [108]. In the paper it was concluded that akebia saponin D could significantly ameliorate the memory deficits and anxiety symptoms. Therefore, it might exert a significant neuroprotective effect on cognitive impairment.

The other studies also proved that akebia saponin D can be a desirable agent to protect against both Alzheimer’s disease-related neuroinflammation and cognitive, including memory, impairment [109]. The protective effect of akebia saponin D was investigated in vivo in rats by bilateral intracerebroventricular injections of amyloid β peptide (Aβ1–42) to induce memory impairment. Moreover, the anti-inflammatory and neuroprotective properties were studied using histochemistry and biochemistry methods.

The dammarane-type triterpenoid saponins identified in *Bacopa monnieri* (L.), including bacoside A also have neuroprotective and memory enhancing properties [110].

There are several in vitro and in vivo studies on the pharmacological properties of *Bacopa monnieri* which highlighted its neuroprotective properties. These abilities are mainly due to its antioxidative, antiapoptotic and anti-inflammatory potential. As shown by the results of the in vivo studies, the extract containing bacopasaponins could mitigate memory impairment and the degeneration of neurons in the hippocampus [111].

Unfortunately, very few systematic structure–activity relationship studies on the mechanism of neuroprotection shown by the triterpenoid saponins have been carried out [112]. Quantitative Structure–Activity Relationship (QSAR) studies are used for investigating the dependence between the structure of a substance and its biological activity. Contemporary drug design, toxicology and environmental monitoring often use the QSAR methodology. One of the main assumptions of QSAR studies is to view biological activity as a sum of the different interactions that a compound undergoes in the reaction with the sites of action (receptors), as well as during transport through biological membranes [113]. Various QSAR models for estimation of the ability of compounds to cross the blood–brain barrier were previously established. Most were based on the steric, electronic and lipophilic properties of the molecules [71,90,114,115,116,117,118,119,120,121,122] as well as on the LFER approach [43].

Studies on the structure–bioactivity relationships, in this case combining the ability of triterpenoid saponins to cross the BBB with their physicochemical parameters, are of significant importance. Therefore, they were intended to bridge a gap in this regard. For this purpose, the analysis of physicochemical parameters, including steric, lipophilic and electronic ones, was made. Taking into account the important physicochemical parameters (Table 4) and the above-mentioned QSAR model (Equation (2)), which proved to be effective in the case of the previously tested saponins [90], the logBB values were determined.

The analysis of the physicochemical parameters, among others, was based on the Hansch approach [58,92] and the Lipinski “rule of five” [95]. The analysis of the obtained values shows that the most lipophilic compound is arjunic acid, and the least is platycodin D. Moreover, platycodin D has the largest molecule (MW = 1225.32 g/mol) and is the most polarizable, in contrast to arjunic acid. Among the tested compounds, arjunic acid has the smallest topological polar surface area value (TPSA = 97.99Å^2^). A TPSA is commonly used in medicinal chemistry for the optimization of compound ability to cross biological membranes. To penetrate the BBB (and thus act on receptors in the CNS), a TPSA should be less than 90 Å^2^ [123]. However, in order to cross any biological barriers, the size of a molecule and its lipophilicity are of crucial importance [58,92]. The analysis of the obtained values shows that arjunic acid is the most lipophilic and has the smallest molecular weight. Therefore, it is largely probable that, among the tested saponins, arjunic acid will cross the blood–brain barrier in the most effective way.

It is commonly known that both computational and biomimetic studies are of enormous significance, particularly in the early stages in the drug discovery process. These methods allow the characterization of already known as well as newly discovered compounds and to predict animal behaviour in the in vivo tests [64,124,125]. However, there are no scientific reports on the triterpenoid saponins’ permeability studies through the BBB using biomimetic chromatographic methods.

As far as is known, this is the first paper on the applicability of the biomimetic chromatographic methods in predicting the penetration of triterpenoid saponins through any biological barriers. In this case, the tested barrier was the blood–brain.

In the stage of biomimetic chromatographic studies there the following methods were applied: High Performance Liquid Chromatography (HPLC) with Immobilized Artificial Membrane (IAM) and cholesterol (CHOL) stationary phases, as well as Bio-partitioning Micellar Chromatography (BMC). All these methods are commonly used to determine permeation of a substance through biological barriers (see Table 1).

The chromatographic biomimetic stationary phases, i.e., IAM and CHOL are considered to be the best chromatographic models used to assess the permeation capacity of substances through specific biological barriers. Solute partitioning between two phases is a well-known phenomenon in which these phases can be two fluid ones (organic/aqueous solvent mixtures), or a suspension of particles in the solute partitions between the continuous phase of the solvent and the surface of the particles [126]. The membrane partition coefficient is defined as the partition coefficient between membrane suspensions and an aqueous phase. Since the membrane partition coefficient correlates with permeability of a substance through biological barriers, it can provide critical insight into the solute–membrane interactions [126]. Unfortunately, it is difficult to measure the membrane partition coefficient in vivo. However, the retention factors (k) obtained using the systems imitating cell membrane (IAM, CHOL, BMC) are directly proportional to the partition coefficients of a solute on the given stationary phase. Therefore, the partition coefficients of analyte between the stationary phase and water can give us some information about ability of a substance to cross specific biological barriers [126].

Based on the obtained IAM and CHOL results, excellent linear relationships between the logarithms of retention factors (logk) and the percentage of organic modifier (acetonitrile) in the mobile phases were found over the whole eluent composition range studied, with correlation coefficient R^2^ equal to 0.982 and 0.991, respectively. For the tested BMC system, the relationships between logk and the micellized surfactant concentration, C_M_, expressed as: C_M_ = C − CMC, where C is the total surfactant concentration, and CMC is the critical micelle concentration, were also obtained with great linearity (R^2^ 0.898). The obtained retention factors were then extrapolated to pure water by extrapolating to the zero acetonitrile concentration in the mobile phases (IAM, CHOL) and to the zero surfactant concentration in the BMC systems. Figure 1 shows that there are no great differences between the obtained logkw values. It is noteworthy that among the lipophilic compounds, i.e., arjunic acid, akebia saponin D, and bacoside A, the highest logkw values were obtained using the CHOL stationary phase. Assuming that the logkw parameter is alternative to the logPow lipophilicity descriptor, it can be noticed that the obtained logkw-CHOL values are mostly similar to the logPow values calculated in silico. In the case of platycodin D, the differences in the logkw values are negligible. This confirms that all three chromatographic methods, i.e., HPLC with both the immobilized artificial membrane and the cholesterol immobilized on silica gel, as well as the bio-partitioning micellar chromatography, described the BBB-permeation potential of triterpenoid saponins in a similar way.

In Figure 2 it can be observed that similar logBB values were obtained for hydrophilic platycodin D applying both the biomimetic and computational methods. The smallest difference in logBB values obtained using biomimetic and computational studies is observed between the logBB-CHOL and logBB-QSAR values (the absolute error is equal to 0.085) whereas the greatest difference is observed between logBB-IAM value and those obtained in silico (the absolute error is 0.393). The greatest differences are those obtained between the logBB values calculated using the biomimetic methods and those in silico in the case of lipophilic compounds. Significant differences are noticeable especially in the case of bacoside A between logBB-CHOL value and those obtained in silico (the absolute error is equal to 2.52). One of the possible explanations is that the computational studies do not take into account the interactions of a compound with a biological membrane that are possible in the cellular environment, e.g., electrostatic interactions [127]. The IAM and CHOL systems used here offer polar heads as the first contact site for solutes whereas the BMC allows the analysis of interactions in a micellar system, for example based on Foley’s equation (Equation (3)) [97], where the interactions in the micellar systems have been characterized.

Knowledge of the type of interaction between the analyte and the micelle, which in this case is a model of the blood-brain barrier, can provide valuable information on the mechanism of interactions between a substance and a barrier. For this purpose, important physicochemical parameters such as the K_MA_, the analyte-micelle association constant, and P_SW_, the partition coefficient of the analyte between the stationary phase and water, have been calculated. Based on the above–mentioned parameters, one can determine the strength of analyte interactions with the biological membrane. In these studies, it was indicated for the first time that the logarithm of the analyte–micelle association constant (logK_MA_) can characterize the passage of substances through the blood–brain barrier expressed by logBB. In other words, the logK_MA_ parameter can be treated as an equivalent to the logBB. Moreover, as shown in Figure 2 the previously established QSAR model also in this case confirmed its applicability and predictability in assessing the ability of triterpenoid saponins to penetrate the BBB.

## 4. Materials and Methods

### 4.1. Chemicals

The analytical standards of arjunic acid, akebia saponin D, bacoside A, and platycodin D were purchased from Sigma Aldich (Sigma Aldrich, St. Louis, MO, USA; p.a.). Acetonitrile, isopropanol and poly-oxyethylene (23) lauryl ether (Brij35) were purchased from Merck (Darmstadt, Germany; p.a.). Citric acid and disodium hydrogen phosphate (Na_2_HPO_4_) were purchased from Sigma Aldich (Sigma Aldrich, St. Louis, MO, USA; p.a.). Distilled water was obtained from the Direct-Q3 UV apparatus (Millipore, Burlington, MA, USA).

### 4.2. Instrumentation

The Shimadzu Vp liquid chromatographic system (Shimadzu, Kyoto, Japan) equipped with LC 10AT pump, SPD 10A UV-Vis detector, SCL 10A system controller, CTO-10 AS chromatographic oven and Rheodyne injector valve with a 20 µL loop was applied in the HPLC measurements.

### 4.3. Chromatographic Conditions

The solutions of analytical standards of the studied triterpenoid saponins were prepared in methanol (Merck, Darmstadt, Germany; p.a.) at a concentration of 1 mg/mL. All the compounds proved to be in the neutral form in solution under the experimental conditions. In the research each system was optimized previously. In the IAM and CHOL studies, acetonitrile-phosphate buffer solutions (0.3; 0.4; 0.5; 0.6 *v*/*v*; pH 7.4) were used as mobile phases. In the case of BMC analysis, the buffered solution of Brij35 (0.075; 0.1; 0.125; 0.15 mol/dm^3^; pH 7.4) was used as the mobile phase with the addition of isopropanol as an organic modifier (7% *v*/*v*). The buffer was prepared from the solutions of Na2HPO4 and citric acid (0.01 mol/dm^3^) and the pH 7.4 value was fixed before the preparation of the mobile phases.

The following stationary phases were employed: IAM.PC.DD2 100 × 4.6 mm i.d., 10 µm (Regis Chemicals Company, Morton Grove, IL, USA); Cosmosil Cholester, 75 × 2 mm i.d., 2.5 µm (Genore, Warsaw, Poland); Purosphere RP-18e (ODS), 125 × 4 mm i.d., 5 µm (Merck, Darmstadt, Germany). The flow rates were established as follows: 1.3 mL/min (IAM), 0.4 mL/min (CHOL), and 1 mL/min (BMC). The tested saponins were detected with UV light at 254 nm. All measurements were made at 25 °C. The dead time values were measured from the non-retained compound (citric acid) peaks. All reported logkw values are the average of at least three independent measurements. The values of peak asymmetry factor were in the acceptable range.

### 4.4. Computer Programs

Within the in silico studies the ACD/Percepta software (version 2012, Advanced Chemistry Development, Inc., Toronto, ON, Canada) was used. Statistical analysis of the obtained results was made using the Minitab 18 Statistical Software (Minitab Inc., State College, PA, USA).

## 5. Conclusions

The main aim of the study was to evaluate the ability of naturally occurring triterpenoid saponins to cross the blood–brain barrier using both computational analysis including QSAR methodology and biomimetic studies using adequate liquid chromatographic techniques. To my knowledge, there are no scientific reports on triterpenoid saponins permeability studies through the BBB using the biomimetic chromatographic methods.

In these studies, it was indicated for the first time that the logarithm of the analyte—micelle association constant (logK_MA_) can characterize the passage of substances through the blood–brain barrier expressed as logBB. Comparing the biomimetic-logBB with the logK_MA_ values obtained using the BMC studies, it can be concluded that these values are comparable with each other. The analyte–micelle association constant is therefore a good descriptor of the ability of triterpenoid saponins to cross the BBB. The applied BMC system adequately reflects the cellular environment and can be successfully used to assess the penetration of substances through the blood–brain barrier. Based on the studies presented in this paper, it is largely probable that three of the four tested saponins, i.e., arjunic acid, akebia saponin D, and bacoside A can penetrate the BBB in contrast to platycodin D which does not cross this barrier. Moreover, the analysis of the obtained values shows that arjunic acid is the most lipophilic and has the smallest molecular weight. Therefore, it is largely probable that, among the tested saponins, arjunic acid will cross the BBB in the most effective way.

The research presented in the paper was also aimed at finding out which physicochemical parameters of the molecule are responsible for the molecule’s ability to cross the blood–brain barrier. Therefore, structure–bioactivity relationships studies were carried out. The influence of specific physicochemical parameters on the ability of the triterpenoid saponins to cross the BBB was investigated. The applicability and predictability of the previously established QSAR model based on logPow, ΔlogP, and E were then confirmed.

The presented research has proved that both the computational and biomimetic tests can be a useful screening tool for assessing the ability of a molecule to cross the specific biological barriers. Due to the ability of the above-mentioned substances to cross the blood–brain barrier, it can be assumed that these substances can be the subject of further research on their supposed neuroprotective properties.

## Figures and Tables

**Table 1:** The use of Bio-partitioning Micellar Chromatography (BMC), Immobilized Artificial Membrane (IAM), and cholesterol (CHOL) chromatography to evaluate the biological activity of organic compounds.

References	Drugs	BMC/IAM System	Biological Activity
[53]	Anticonvulsant drugs	Brij 35: 0.02 M; 0.04 M; 0.06 M; pH: 7.4	Anticonvulsant properties
[52]	Non-steroidal anti-inflammatory drugs	Brij 35: 0.02 M; 0.04 M; 0.06 M; pH: 7.4	Anesthetic potency
[48]	Local anesthetics	Brij 35: 0.02 M; 0.04 M; 0.06 M; pH: 7.4	Anesthetic potency
[72]	Barbiturates	Brij35: 0.02 M; 0.04 M; 0.06 M SDS: 0.05 M; 0.1 M; 0.15 M CTAB: 0.01 M; 0.02 M; 0.05 M pH: 3.5 and 7.4	Hypnotic activity
[73]	Catecholamines	SDS: 0.05 M; 0.1 M + MeOH, EtOH, 1-propanol, pentanol pH: 2–7	β- andrenergic activity
[74]	Benzodiazepines	Brij35: 0.02 M; 0.04 M; 0.06 M pH: 7.4	Toxicity and anxiolytic activity
[51]	Phenothiazines	Brij35: 0.02 M; 0.04 M; 0.06 M pH: 7.4	Pharmacokinetics, preclinical pharmacology, and therapeutic efficacy parameters; antipsychotic potential
[45]	Structurally diverse drugs	Brij35: 0.04 M pH: 7.4 and 6.5	Oral absorption
[75]	Fatty acids and polyphenols	Brij35: 0.04 M; 0.06 M; 0.08 M; 0.1 M; 0.12 M + acetonitrile CTAB: 0.04 M; 0.06 M; 0.08 M; 0.1 M; 0.12 M + acetonitrile SDS: 0.04 M; 0.06 M; 0.08 M; 0.1 M; 0.12 M + acetonitrile, dioxane, tetrahydrofuran, acetone pH: 7.4	Oral, jejunum and Caco-2 absorption
[76]	Structurally diverse drugs	Brij35: 0.04 M pH: 7.4	BBB permeability
[77]	Phenols	Brij35: 0.06 M; 0.08 M; 0.1 M; 0.12 M + isobutanol (5% *v*/*v*) pH: 7.4	BBB permeability
[78]	Non-steroidal anti-inflammatory drugs	Brij35: 0.04 M pH: 3.5–8	Skin permeability
[79]	Fatty acids and polyphenols	Brij35: 0.04 M; 0.06 M; 0.08 M; 0.1 M; 0.12 M + acetonitrile CTAB: 0.04 M; 0.06 M; 0.08 M; 0.1 M; 0.12 M + acetonitrile SDS: 0.04 M; 0.06 M; 0.08 M; 0.1 M; 0.12 M + acetonitrile, dioxane, tetrahydrofuran, acetone pH: 7.4	Percutaneous absorption
[80]	Anxiolytics, antihistamines, β-blockers, antiepileptics, antipsychotics	SDS: 0.07 M; 0.09 M pH: 7.4	Protein drug binding properties
[81]	Structurally diverse drugs	PBS or PBS-acetonitrile: 5–25% *v*/*v* pH: 7.4	Cell permeability, human oral absorption, % plasma protein binding
[82]	Novel β-hydroxy-β-aryl-alkanoic acids	Brij35: 0.04 M pH: 7.4	Gastrointestinal absorption
[83]	Structurally diverse drugs	Brij35: 0.04 M pH 7.4	Blood to lung; blood to liver; blood to fat; blood to skin partition coefficients
[84]	Newly-synthesized 17-β-carboxamide steroids	Brij35: 0.04 M pH: 5.5 and 7.5	Skin and corneal permeability
[85]	Structurally diverse drugs	Brij35: 0.04 M pH: 7.4–7.7	Ocular tissue permeability
[86]	Structurally diverse drugs	Brij35: 0.04 M pH: 7.4	BBB permeability
[87]	Benzophenone ultraviolet filters	Brij35: 0.01 M; 0.02 M; 0.03 M pH: 7.4 and 6.5	Ecotoxicity and skin permeability
[65]	Structurally diverse pesticides	Phosphate-buffered saline (PBS) or PBS-acetonitrile: 5–25% *v*/*v* pH: 7.4	Ecotoxicity
[61]	Structurally diverse compounds	Buffer- MeOH: 70:30 *v*/*v* pH: 7.4	BBB permeability
[66]	Structurally diverse drugs	PBS or PBS-acetonitrile: 5–25% *v*/*v* pH: 7.4	Bioconcentration factor
[88]	Structurally diverse drugs	Acetonitrile-buffer pH: 7.4	Interactions between the solutes and the immobilized phospholipid membranes
[67]	Structurally diverse drugs	Acetonitrile-buffer: 0–30% *v*/*v* pH: 7.4	Human oral absorption
[63]	Newly-synthesized drug-like compounds	Acetonitrile-buffer pH 7.4	Blood–brain barrier permeation
[89]	Newly synthesized antiproliferative and analgesic active compounds	Acetonitrile-buffer pH 7.4	Lipophilicity

**Table 2:** The chemical structures of the tested compounds.

Name	Structure
Arjunic acid	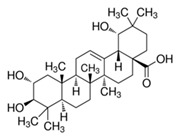
Akebia saponin D	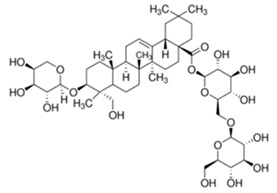
Bacoside A	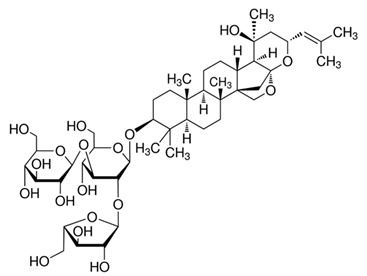
Platycodin D	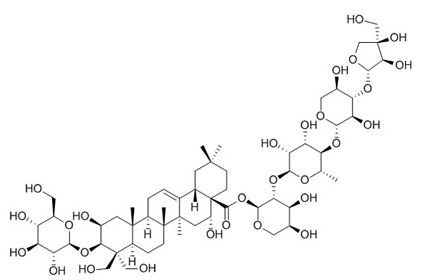

**Table 3:** The pharmacokinetic BBB descriptors calculated in silico (ACD/Percepta).

Name	logBB	logPS	logPS_Fubrain_	Fu	Fb
Arjunic acid	0.14	−3.2	−4.9	0.012	0.02
Akebia saponin D	0.32	−4.4	−5.7	0.12	0.06
Bacoside A	0.03	−3.6	−4.5	0.14	0.13
Platycodin D	<−2	−3.6	−9.0	0.52	0.98

logBB, Blood–brain barrier penetration descriptor; logPS, Logarithmic permeability–surface areaproduct; log(PS_Fubrain_), Brain/plasma equilibration rate; Fu, Fraction unbound in plasma; Fb, Fraction unbound in the brain.

**Table 4:** The chosen physicochemical parameters determined for the tested saponins.

Name	LogPow (Octanol/Water)	logPhw (Heptane/Hater)	logPcw (Cyclohexane /Water)	Molecular Weight (MW) (g/mol)	Topological Polar Surface Area (TPSA) (Å^2^)	Polarizability
Arjunic acid	5.2	4.179	4.029	488.70	97.99	54.15
Akebia saponin D	0.8	−9.919	−9.932	929.10	294.98	91.23
Bacoside A	2.8	−7.553	−7.110	768.97	215.83	78.66
Platycodin D	−3.7	−24.512	−24.704	1225.32	453.28	114.32

## Supplementary Materials

The following are available online at https://www.mdpi.com/article/10.3390/ijms22073573/s1, Table S1: Experimentally obtained logBB [91] values for the compounds used as the training and test sets in the QSAR studies; Table S2: The LFER and chosen physicochemical parameters, calculated for the entire set of the tested compounds (ACD/Percepta).
