# Supplementary material for: Biomimetic Chromatographic Studies Combined with the Computational Approach to Investigate the Ability of Triterpenoid Saponins of Plant Origin to Cross the Blood–Brain Barrier
Source: Int J Mol Sci. 2021 Mar 30;22(7):3573. doi: 10.3390/ijms22073573 (PMC8037809; doi:10.3390/ijms22073573)
Supplement: Supplementary file 1 [file ijms-22-03573-s001.zip › Table S1.docx]

| No. | CAS No. | logBB |
| --- | --- | --- |
| 1 | 30516-87-1 | –0.72 |
| 2 | 73590-58-6 | –0.82 |
| 3 | 101-40-6 | 1.08 |
| 4 | 23830-88-8 | 0.16 |
| 5 | 103-90-2 | –0.74 |
| 6 | 60-80-0 | –0.07 |
| 7 | 54910-89-3 | 1.08 |
| 8 | 54739-18-3 | 0.79 |
| 9 | 79559-97-0 | 1.6 |
| 10 | 53179-11-6 | 0.77 |
| 11 | 7481-89-2 | –1.5 |
| 12 | 161814-49-9 | –0.56 |
| 13 | 69655-05-6 | –1.28 |
| 14 | 129618-40-2 | 0 |
| 15 | 159989-64-7 | –0.93 |
| 16 | 151-83-7 | –0.06 |
| 17 | 76-73-3 | 0.2 |
| 18 | 76-75-5 | –0.45 |
| 19 | 59468-90-5 | –0.07 |
| 20 | 1088-11-5 | 0.61 |
| 21 | 439-14-5 | 0.56 |
| 22 | 13655-52-2 | –0.23 |
| 23 | 29122-68-7 | –1 |
| 24 | 63659-18-7 | 0.39 |
| 25 | 120014-06-4 | 0.89 |
| 26 | 357-70-0 | 0.32 |
| 27 | 123441-03-2 | 0.88 |
| 28 | 142852-50-4 | 1.14 |
| 29 | 91374-21-9 | 0.25 |
| 30 | 52-26-6 | –0.16 |
| 31 | 83903-06-4 | –1.06 |
| 32 | 59-33-6 | 0.49 |
| 33 | 83-67-0 | –0.29 |
| 34 | 36318-56-6 | 0.04 |
| 35 | 4201-26-7 | 0.14 |
| 36 | 38941-33-2 | 0.58 |
| 37 | 76-57-3 | 0.08 |
| 38 | 66357-35-5 | 0.32 |
| 39 | 82626-48-0 | 0.12 |
| 40 | 133099-04-4 | 0.06 |

Table S1. Experimentally obtained logBB [91] values for the compounds used as the training and test sets in the QSAR studies*.
