# Supplementary material for: Biomimetic Chromatographic Studies Combined with the Computational Approach to Investigate the Ability of Triterpenoid Saponins of Plant Origin to Cross the Blood–Brain Barrier
Source: Int J Mol Sci. 2021 Mar 30;22(7):3573. doi: 10.3390/ijms22073573 (PMC8037809; doi:10.3390/ijms22073573)
Supplement: Supplementary file 1 [file ijms-22-03573-s001.zip › Table S2.docx]

Table S2. The LFER and chosen physicochemical parameters, calculated for the entire set of the tested compounds (ACD/Percepta).

| **No.** | **CAS No.** | **A** | **B** | **S** | **E** | **V** | **MW** | **TPSA** | **logP_o/w_** | **Polarizability** | **logP_c/w_** | **logP_h/w_** | **ΔlogP** |
| --- | --- | --- | --- | --- | --- | --- | --- | --- | --- | --- | --- | --- | --- |
| 1 | [84680-75-1](https://www.chemsrc.com/en/baike/895796.html) | 1.72 | 4.87 | 4.62 | 3.72 | 6.4335 | 869.04 | 240.36 | 5.02 | 86.01 | –7.125 | –7.543 | 12.145 |
| 2 | [84676-89-1](https://www.chemsrc.com/en/baike/402120.html) | 1.99 | 4.73 | 4.57 | 3.86 | 5.8542 | 798.95 | 234.29 | 3.47 | 78.53 | –8.445 | –8.9 | 11.915 |
| 3 | 84687-42-3 | 2.33 | 4.69 | 4.4 | 3.97 | 5.8385 | 784.97 | 228.22 | 3.767 | 78.41 | –9.269 | –9.533 | 13.036 |
| 4 | 84687-43-4 | 2.26 | 4.71 | 4.38 | 3.98 | 5.8385 | 784.97 | 228.22 | 3.757 | 78.41 | –9.015 | –9.3 | 12.772 |
| 5 | [84687-44-5](https://www.chemsrc.com/en/baike/75310.html) | 3.09 | 6.12 | 5.52 | 4.92 | 6.8688 | 947.11 | 307.37 | 2.093 | 91.78 | –15.628 | –15.861 | 17.721 |
| 6 | 84687-45-6 | 3.01 | 6.22 | 5.46 | 4.96 | 6.8688 | 947.11 | 307.37 | 1.899 | 91.78 | –15.592 | -15.823 | 17.491 |
| 7 | [84687-46-7](https://www.chemsrc.com/en/baike/743281.html) | 3.03 | 6.15 | 5.5 | 4.93 | 6.8688 | 947.11 | 307.37 | 2.083 | 91.78 | –15.412 | –15.661 | 17.495 |
| 8 | 30516-87-1 | 0.47 | 1.7 | 1.77 | 1.62 | 1.8192 | 267.24 | 109.19 | 0.171 | 0 | –3.401 | –3.625 | 3.572 |
| 9 | 73590-58-6 | 0.35 | 2.05 | 3.18 | 2.67 | 2.5161 | 345.42 | 96.31 | 1.444 | 37.27 | –1.995 | -2.926 | 3.439 |
| 10 | 101-40-6 | 0.13 | 0.53 | 0.43 | 0.37 | 1.5088 | 155.28 | 12.03 | 3.733 | 19.75 | 3.485 | 3.554 | 0.248 |
| 11 | 23830-88-8 | 0.42 | 1.04 | 1.14 | 1.5 | 1.5317 | 230.09 | 36.42 | 1.987 | 22.71 | –0.264 | –0.376 | 2.251 |
| 12 | 103-90-2 | 0.91 | 0.93 | 1.66 | 1.12 | 1.1724 | 151.16 | 49.33 | 0.149 | 16.81 | –4.518 | –4.582 | 4.667 |
| 13 | 60-80-0 | 0 | 1.28 | 1.75 | 1.42 | 1.4846 | 188.23 | 23.55 | 0.24 | 21.63 | –1.228 | –1.691 | 1.468 |
| 14 | 54910-89-3 | 0.13 | 0.78 | 1.19 | 1.01 | 2.24 | 309.33 | 21.26 | 5.147 | 31.67 | 4.728 | 4.46 | 0.419 |
| 15 | 54739-18-3 | 0.23 | 1.14 | 0.95 | 0.66 | 2.3113 | 318.33 | 56.84 | 4.267 | 30.46 | 3.083 | 3.056 | 1.184 |
| 16 | 79559-97-0 | 0.13 | 0.67 | 1.44 | 1.83 | 2.2647 | 306.23 | 12.03 | 5.888 | 34.02 | 5.705 | 5.222 | 0.183 |
| 17 | 53179-11-6 | 0.31 | 1.88 | 2.9 | 2.76 | 3.7697 | 477.04 | 43.78 | 6.431 | 54.52 | 4.235 | 3.349 | 2.196 |
| 18 | 7481-89-2 | 0.44 | 1.9 | 1.78 | 1.63 | 1.506 | 211.22 | 88.15 | –1.653 | 20.19 | –5.618 | –5.815 | 3.965 |
| 19 | 161814-49-9 | 0.64 | 2.61 | 3.52 | 2.71 | 3.8194 | 505.63 | 139.57 | 3.321 | 53.37 | –1.597 | –2.428 | 4.918 |
| 20 | 69655-05-6 | 0.31 | 1.77 | 1.85 | 2.03 | 1.5951 | 236.23 | 88.74 | –0.716 | 22.67 | –3.887 | –4.24 | 3.171 |
| 21 | 129618-40-2 | 0.42 | 1.37 | 2.29 | 2.36 | 1.9446 | 266.3 | 58.12 | 1.83 | 29.23 | –1.059 | –1.667 | 2.889 |
| 22 | 159989-64-7 | 1.27 | 2.81 | 3.62 | 3.2 | 4.5367 | 567.78 | 127.2 | 5.66 | 64.37 | –1.292 | –1.941 | 6.952 |
| 23 | 151-83-7 | 0.24 | 1.42 | 1.55 | 1.27 | 2.0903 | 262.3 | 66.48 | 2.201 | 27.68 | 0.175 | –0.087 | 2.026 |
| 24 | 76-73-3 | 0.52 | 1.3 | 1.41 | 1.11 | 1.8945 | 238.28 | 75.27 | 1.971 | 24.68 | –1.018 | –1.108 | 2.989 |
| 25 | 76-75-5 | 0.51 | 1.34 | 2 | 1.49 | 1.9014 | 242.34 | 90.29 | 1.45 | 25.99 | –1.838 | –2.195 | 3.288 |
| 26 | 59468-90-5 | 0.31 | 1.21 | 1.96 | 2.64 | 2.3215 | 341.77 | 49.89 | 4.219 | 35.95 | 2.491 | 1.893 | 1.728 |
| 27 | 1088-11-5 | 0.47 | 0.88 | 1.94 | 2.05 | 1.933 | 270.71 | 41.46 | 3.435 | 29.97 | 1.115 | 0.616 | 2.320 |
| 28 | 439-14-5 | 0 | 0.94 | 1.95 | 2.07 | 2.0739 | 284.74 | 32.67 | 3.784 | 32.08 | 3.27 | 2.571 | 0.514 |
| 29 | 13655-52-2 | 0.29 | 1.36 | 1.12 | 1.18 | 2.1587 | 249.35 | 41.49 | 3.143 | 29.75 | 1.346 | 1.263 | 1.797 |
| 30 | 29122-68-7 | 0.78 | 1.85 | 1.97 | 1.48 | 2.1763 | 266.34 | 84.58 | 0.839 | 29.44 | –3.963 | –4.105 | 4.802 |
| 31 | 63659-18-7 | 0.29 | 1.53 | 1.31 | 1.31 | 2.5745 | 307.43 | 50.72 | 3.979 | 35.25 | 2.145 | 1.995 | 1.834 |
| 32 | 120014-06-4 | 0 | 1.5 | 2.49 | 2.12 | 3.0307 | 379.49 | 38.77 | 5.024 | 43.77 | 4.121 | 3.282 | 0.903 |
| 33 | 357-70-0 | 0.31 | 1.45 | 1.92 | 1.89 | 2.1734 | 287.35 | 41.93 | 2.375 | 31.84 | 0.011 | –0.431 | 2.364 |
| 34 | 123441-03-2 | 0 | 1.23 | 1.47 | 1.05 | 2.1176 | 250.34 | 32.78 | 2.915 | 28.99 | 2.096 | 1.75 | 0.819 |
| 35 | 142852-50-4 | 0.13 | 1.3 | 2.12 | 2.19 | 3.154 | 376.53 | 32.34 | 6.551 | 45.53 | 5.762 | 5.073 | 0.789 |
| 36 | 91374-21-9 | 0.41 | 1.27 | 1.41 | 1.38 | 2.2321 | 260.37 | 32.34 | 3.51 | 31.07 | 1.298 | 1.113 | 2.212 |
| 37 | 52-26-6 | 0.5 | 1.47 | 1.59 | 2.23 | 2.0648 | 285.34 | 52.93 | 2.471 | 30.93 | –0.426 | –0.694 | 2.897 |
| 38 | 83903-06-4 | 0.4 | 2.57 | 2.2 | 2.55 | 3.1776 | 413.54 | 108.06 | 2.512 | 46.52 | –1.054 | –1.461 | 3.566 |
| 39 | 59-33-6 | 0 | 1.45 | 1.73 | 1.66 | 2.3868 | 285.38 | 28.6 | 3.353 | 34.67 | 2.433 | 1.947 | 0.920 |
| 40 | 83-67-0 | 0.24 | 1.22 | 1.89 | 1.46 | 1.2223 | 180.16 | 67.23 | –0.635 | 17.86 | –3.184 | –3.61 | 2.549 |

A, B, S, E, V: LFER parameters,MW: Molecular weight, TPSA: Topological polar surface area, logP_o/w_: Logarithm of the n-octanol/water partition coefficient, logP_c/w_: Logarithm of the cyclohexane/water partition coefficient, logP_h/w_: Logarithm of heptane/water partition coefficient, ΔlogP: difference between the n-octanol/water and cyclohexane/water logP values.
